# Detection and quantification of renal fibrosis by computerized tomography

**DOI:** 10.1371/journal.pone.0228626

**Published:** 2020-02-13

**Authors:** Eric P. Cohen, John D. Olson, Janet A. Tooze, J. Daniel Bourland, Greg O. Dugan, J. Mark Cline

**Affiliations:** 1 Department of Medicine, University of Maryland Baltimore School of Medicine, Baltimore, Maryland, United States of America; 2 Baltimore Veterans Affairs Medical Center, Baltimore, Maryland, United States of America; 3 Department of Comparative Medicine, Wake Forest University, Wake Forest, North Carolina, United States of America; Northwestern University Feinberg School of Medicine, UNITED STATES

## Abstract

**Objectives:**

Reliable biomarkers for renal fibrosis are needed for clinical care and for research. Existing non-invasive biomarkers are imprecise, which has limited their utility.

**Methods:**

We developed a method to quantify fibrosis by subject size-adjusted CT Hounsfield units. This was accomplished using CT measurements of renal cortex in previously irradiated non-human primates.

**Results:**

Renal cortex mean CT Hounsfield units that were adjusted for body size had a very good direct correlation with renal parenchymal fibrosis, with an area under the curve of 0.93.

**Conclusions:**

This metric is a promising and simple non-invasive biomarker for renal fibrosis.

## Introduction

Fibrosis is an endpoint and agent of renal injury [1], but the mechanisms and timeline of the fibrotic process following tissue injury are still under investigation. Anti-fibrotic therapies are of substantial interest in renal injury research, because their success could improve clinical outcomes. Identifying novel biomarkers of fibrosis could guide the timing of administration of anti-fibrotic therapies and could be used as surrogates of response to therapies. Additionally, identifying and quantifying fibrosis using non-invasive imaging methods is particularly desirable [2].

Non-invasive imaging modalities include ultrasound, magnetic resonance imaging (MRI), and computed tomography (CT). Ultrasound enables a binary assessment of the presence of fibrosis, but kidneys are generally not echogenic until there has been significant loss of function. MRI provides indirect measures of fibrosis, but it is expensive and cannot be used in subjects that have metal in their bodies.

A review of the literature did not find reports of the use of CT to detect or quantify renal fibrosis. The absence of reports in which CT is used to quantify renal fibrosis may be due to the presence of a size-dependent artifact caused by beam hardening. CT numbers, i.e. Hounsfield units, are likely to depend on subject size due to beam hardening effects that are not removed by beam filter and reconstruction algorithms. Briefly, when polychromatic x-ray beams pass through tissue, lower energy photons are absorbed or scattered first. This increases the average energy of the photons in the remaining beam. The absorption coefficient of tissue is energy dependent. This effect causes CT number to decrease as subject size increases [3], and therefore a subject size correction must be performed if CT is to be used to quantitatively measure fibrosis.

Healthy renal tissue (without the use of exogenous contrast) has a CT number of 20 to 40 Hounsfield units (HU) [4]. Tendons, which are made of mostly collagen and water, have a CT number of 75 to 115 HU [5]. Because collagen is a major component of fibrotic tissue, it is reasonable to use these reported CT number values for normal kidney tissue and collagen to predict, based on an area weighted average CT number, that a kidney that is 5% fibrosis by volume will have a mean CT number value that is 10% greater than the CT number of a kidney with no fibrosis. Published coefficients of variation for quantitative CT values are typically in the 2 to 4% range [6]. Thus, CT should be able to detect fibrosis that is more than 5% of the tissue.

We therefore hypothesize that CT scans corrected for subject size can be used to detect and determine the severity of renal fibrosis.

## Methods

A colony of previously irradiated rhesus macaque non-human primates (NHP) is maintained at Wake Forest University. Animals in this study were irradiated at different institutions as part of prior acute radiation studies. At the end of their respective acute radiation studies, animals were transferred to Wake Forest to become part of the Radiation Survivor Cohort so that the longer term delayed effects of radiation exposure could be studied. As reported in Hale et al [7], the animals in this study were irradiated at Wake Forest University, Armed Forces Radiobiology Research Institute (AFRRI), University of Maryland, and University of Illinois. Details of their irradiation are in Table 1 of the supplement. All animal procedures were performed under a program accredited by the Association for Assessment and Accreditation of Laboratory Animal Care and approved by the Wake Forest University Animal Care and Use Committee (Animal Welfare Assurance Number A-3391-01). This program is in accordance with the Animal Welfare Act and the Guide for the Care and Use of Laboratory Animals (National Research Council. 2011). Animals were monitored twice daily by laboratory staff for any signs of illness, injury or disease. Daily assessments included activity level, fecal output, any signs of edema, vomiting, hemorrhage, seizure activity, respiratory distress, and food intake. These assessments were performed a minimum of four hours apart and once daily on weekends. Animals were fed a diet that mimics the North American Diet (5LOP, LabDiet, St. Louis, MO) with the addition of fruit and vegetables and a variety of daily enrichments.

Toxicity is assessed according to the Children’s Cancer Group Clinical Toxicity Criteria, which is added as supplementary material. Euthanasia is achieved by anesthetizing the animal to a deep surgical plane of anesthesia with sodium pentobarbital (dose is typically 20–30 mg/kg i.v.) and exsanguination by perfusion via the left ventricle with cold NaCl or LRS solution (1-2L).

Twenty-two animals (20 male, 2 female) were included in this study, 10 NHP (9 male, 1 female) that had received 5 to 8 Gy total body irradiation 6.4 to 12 years previously, 2 age matched non-irradiated controls (1 male, 1 female), and 10 male NHPs that received 10 Gy chest only irradiation; they had minimal renal radiation exposure. These ten have been analyzed for other studies [8]. As reported therein, the kidneys of chest-only-irradiated NHP received between 1% and 5% of the intended 10 Gy dose, or between 0.1 and 0.5 Gy.

These 22 animals were selected because each animal had a single full body computerized tomography (CT) scan, without the use of intra-venous contrast, within seven months before death and had one or both kidneys preserved for histology.

CT scanning was performed on anesthetized animals using a 32 slice Toshiba Aquilion Scanner (Tustin, CA) at 120 kV, 300 mA, field of view (FOV) = 320 mm, matrix = 512x512, and slice thickness of 0.5 mm. This CT scanner had monthly quality assurance performed to confirm that the stability of the CT number for water was 0 +/- 3 HU.

**Fig 1:**
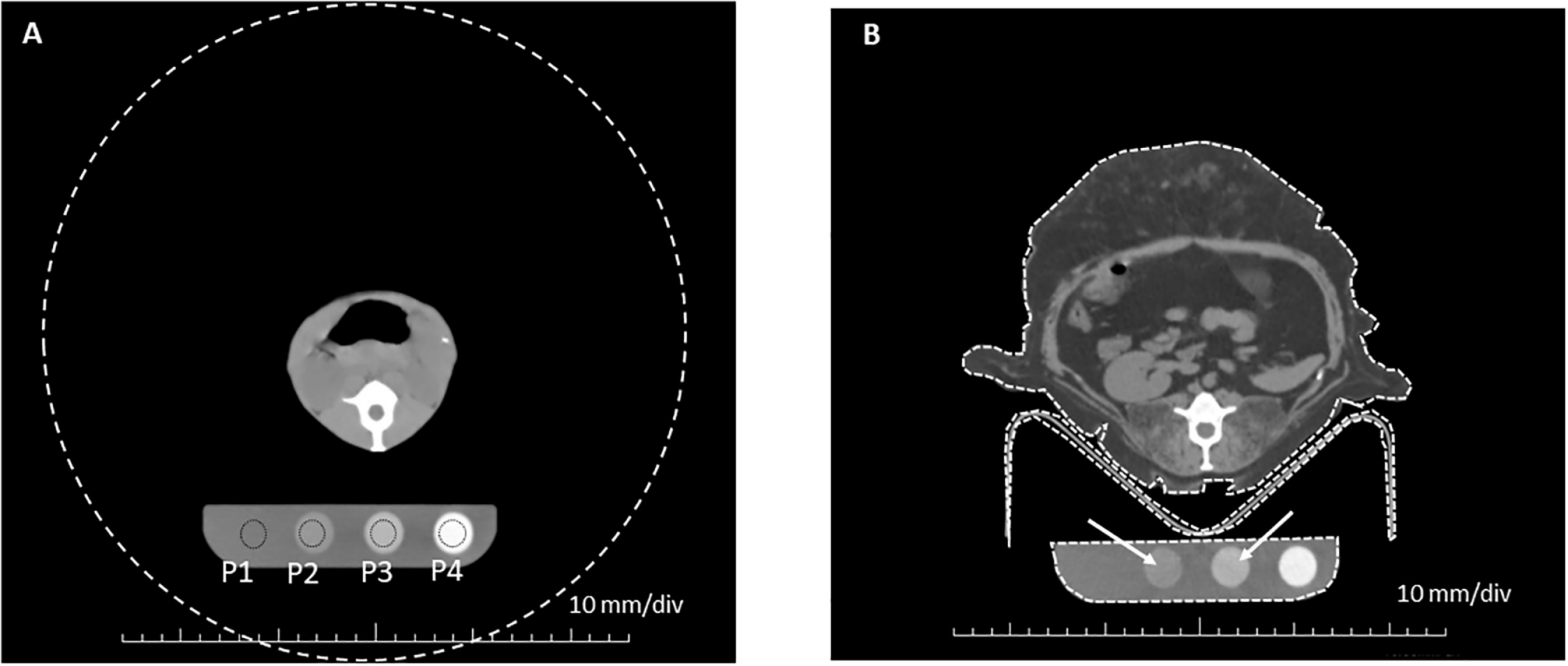
Regions of interest and total signal index. A) Axial image at the level of the right kidney of a small animal in the study. Black circles denote the four phantom ROIs that were traced (P1-P4). The large dashed white circle is the field of view (FOV) of the scan. The area and mean CT# of the FOV ROI were used to calculate the total CT signal index. B). Axial image at the level of the right kidney of a large animal in the study. White dashed lines show an example of the ROIs that were hand traced to measure the total cross sectional area of tissue and material in the slice. This particular slice was not used in the phantom study because of the presence of the trough. The trough shadow artifact in P3 can be seen (white arrows). This slice was selected for the figure because it is at the same anatomical level as the slice for the smaller animal in Fig 1A.

**Fig 2:**
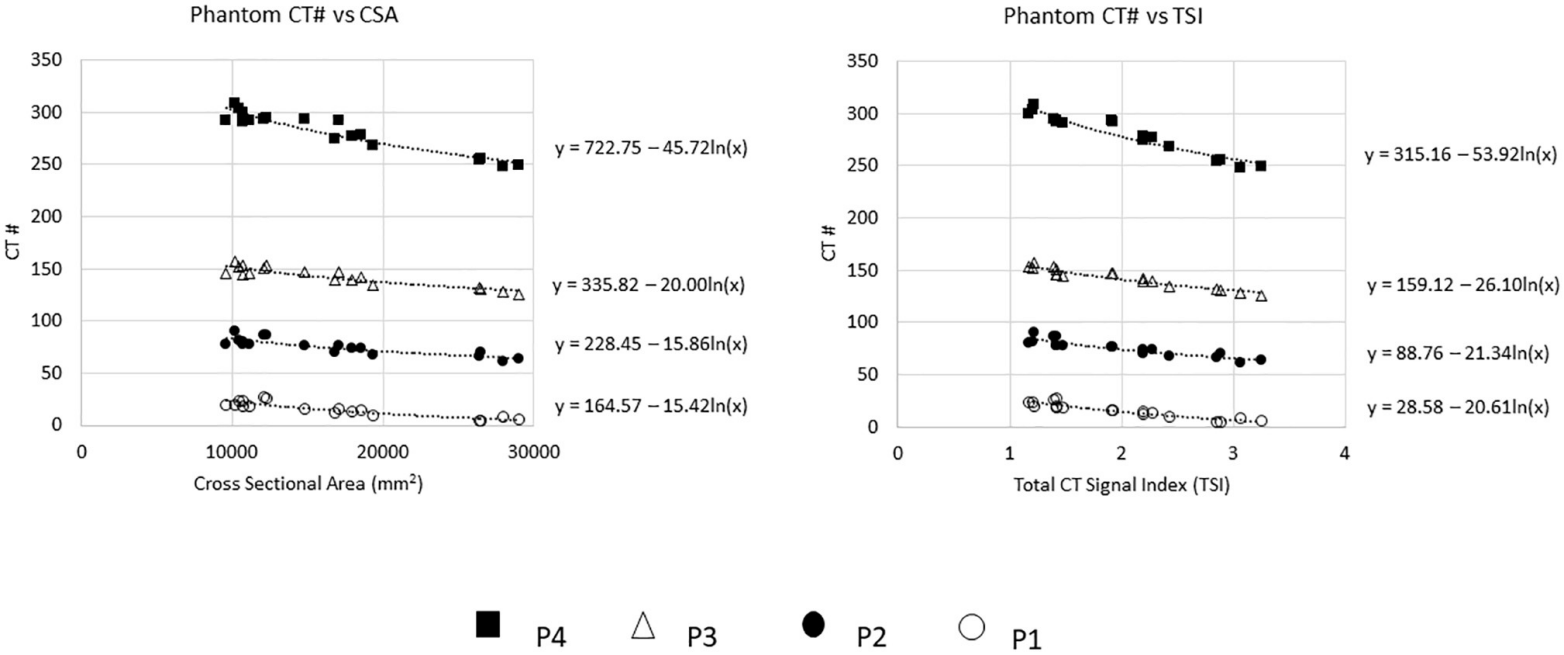
CT numbers and total signal index. Phantom ROI CT# versus CSA(left) and TSI(right) with regression of ln(size index). These plots show that CT# decreases as subject size increases in phantom ROIs of different densities. For this scanner, one obtains an equation for the relationship between CT# and subject size of the form: CT# = B–A ln(size index) (Equation 1). The constant coefficient B in the equation 1 is equal to the CT# of the phantom ROI when the slice has a size index of 1. The magnitude of the coefficient in the ln term (A) increases as the density of the phantom increases. Akaike’s Information Criterion (AIC) was used to compare the fit of CT# vs CSA to the fit of CT# vs TSI. TSI explained more size dependent variation in CT number than area, as evidenced by the lower AIC (AIC = 380.6 for CSA vs 360.5 for TSI).

Nine of the 22 animals were used in a phantom-calibrated study to characterize our scanner’s residual beam hardening artifact. These 9 animals were selected because each animal had the calibration phantom in the scan and also had at least two slices that included the phantom and did not include a trough that was used to position the animals. The trough was avoided for this phantom study because it created streak artifacts in the image of the phantom. The phantom was a tissue neutral quantitative CT (QCT) phantom with 4 regions corresponding to 0, 50, 100, and 200 mg/ml of calcium hydroxyapatite. (Image Analysis, Inc, Columbia, KY). Circular regions of interest (ROI) were drawn in the four cylinders of the phantom using TeraRecon image analysis software. (Durham, NC) (Fig 1). Two indexes of animal size were considered; 1) the total cross sectional area (CSA) of the x-ray absorbing animal tissue in the slice was measured., and 2) the total CT signal in the slice was calculated by tracing a ROI that contained the entire field of view (FOV). The total CT signal in a slice was defined as the mean CT number for the entire FOV times the total area of the FOV. A total signal index (TSI) was calculated by subtracting the CT number for air (-1024 for this scanner) from the mean CT number for the entire FOV. This ensured that the TSI would be a positive number. Then the mean CT number (-1024) was multiplied times the total area of the FOV in mm^2^. This value was divided by 10^7^. This yielded TSI values between 0 and 10 for our cohort. The CT numbers of the phantom ROIs were plotted as a function of each size index (Fig 2). The relationship between CT number and subject size for our scanner was modeled by regressing CT number for each phantom on the natural log of the TSI. These equations were used to adjust for beam hardening as described in the accompanying supplement.

**Fig 3:**
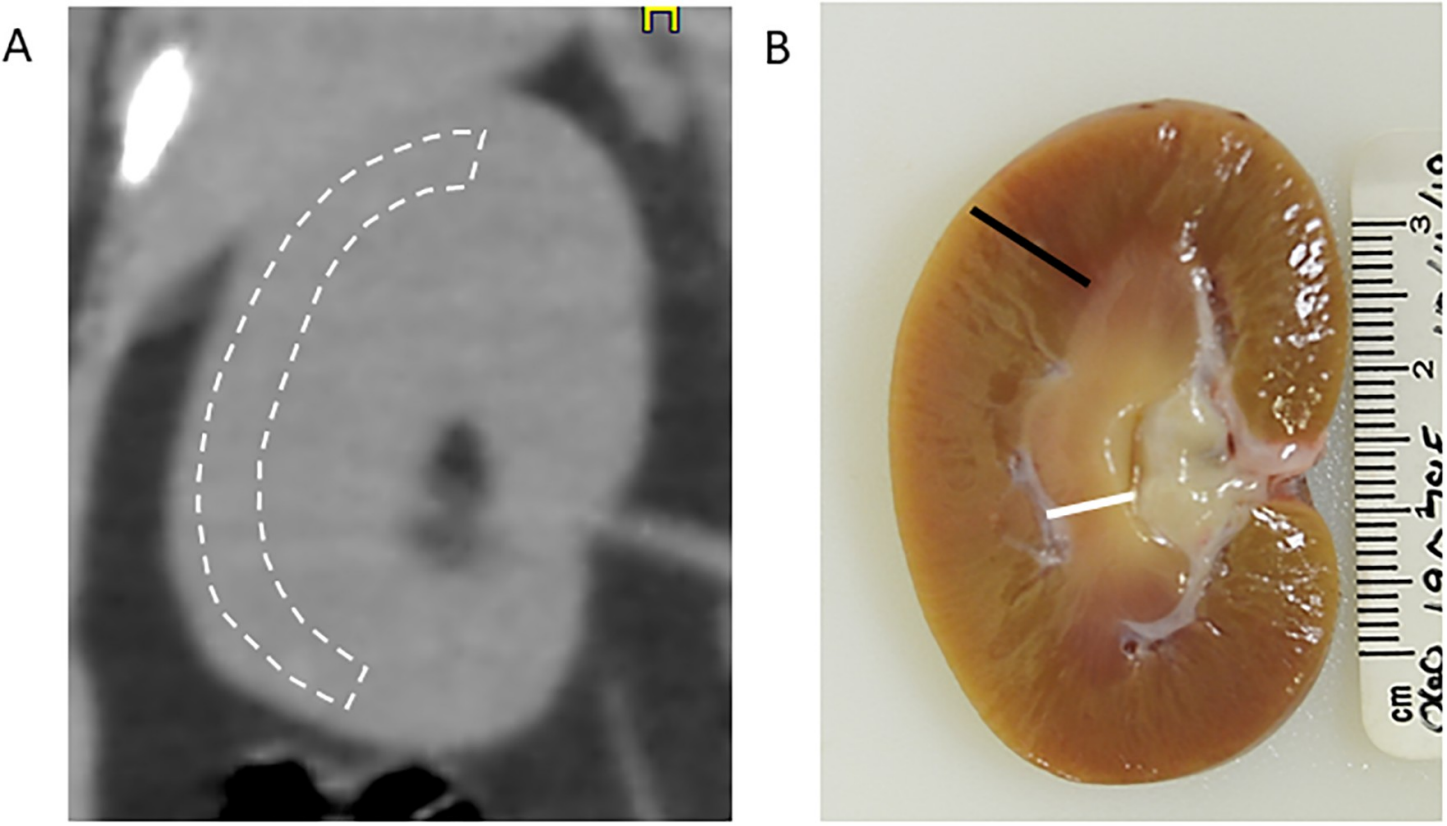
Regions of interest and kidney anatomy. A) Renal Cortex ROIs were manually traced in three slices of a coronal reconstruction of each CT data set to get the average CT number of the renal cortex. B) Shows a sagittal view of a rhesus monkey kidney. A black line indicates the cortex and a white line indicates the medulla.

For all study subjects, CT images were analyzed by a single researcher who was masked to the fibrosis score of the kidneys. Renal cortex regions of interest (ROI) were hand traced in three coronal slices and averaged to get a mean CT number value for that kidney’s cortex. The ROIs excluded medulla based on the anatomy of NHP kidneys (Fig 3). They did not include cystic areas. There was no case showing hydrophrosis. Indexes of animal size were measured in three axial slices that intersected the renal cortex ROIs. The size indexes of these three measurements were averaged to yield the animal size index for each NHP.

Blood samples were obtained just before euthanasia and tested for blood urea nitrogen (BUN) using commercial kits. The normal range in NHP is 19 to 24 mg/dl [9]. Kidneys were formalin fixed, paraffin embedded and sliced into 4 micron thick whole-organ sections, two to four per animal. Sections were deparaffinized and hydrated to water, post fixed in Bouin’s fixative overnight at room temperature, then washed and stained with Masson Trichrome.

**Fig 4:**
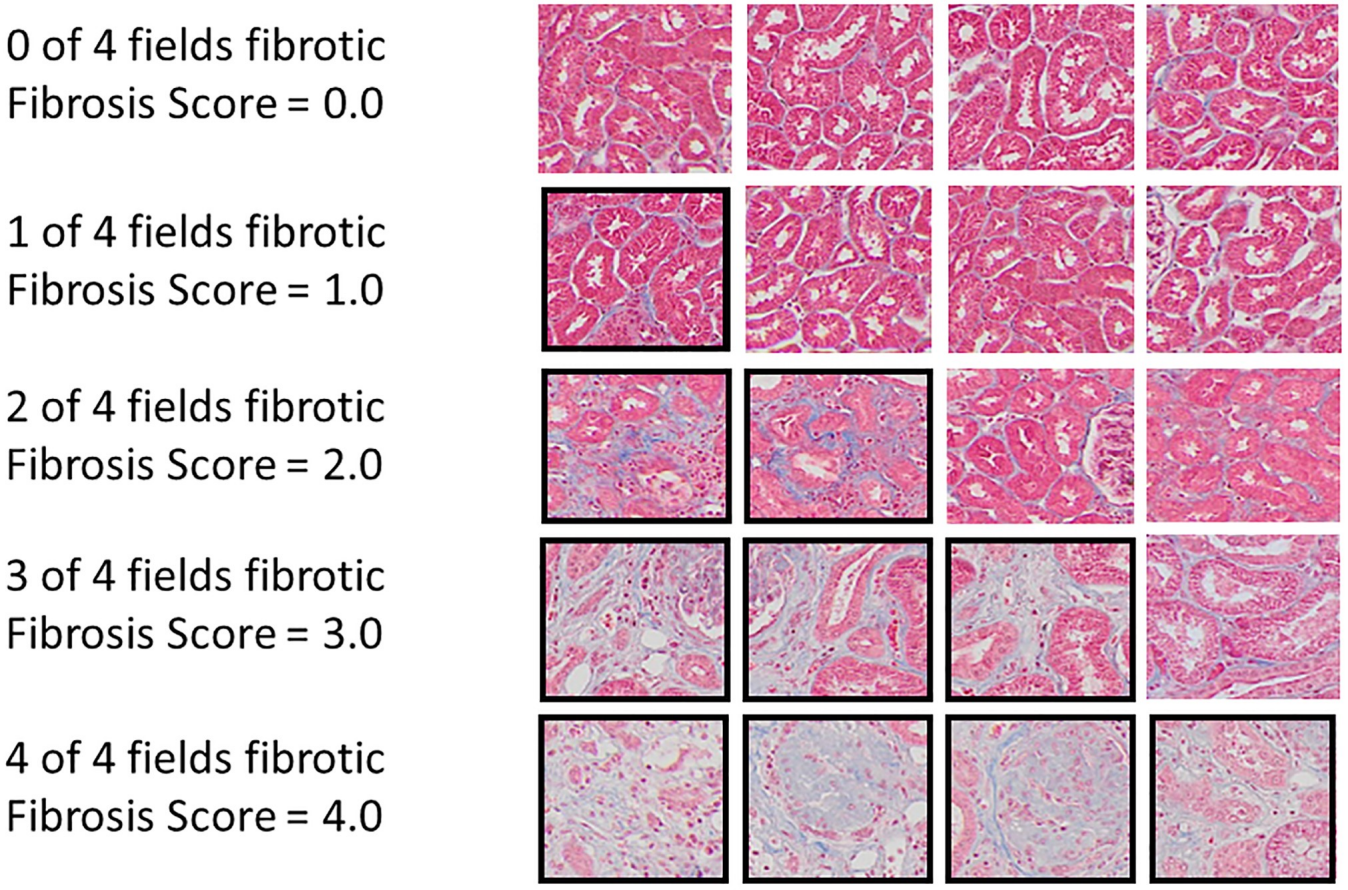
Histologic fibrosis scoring. Microscope fields at 100 x magnification of Masson’s Trichrome stained kidney sections representing fibrosis scores of 1 to 4. Collagen is stained blue.

Cortical fibrosis was scored twice for each kidney in a masked fashion, on a scale of 0 through 4, by assessment of at least four microscopic fields at 100x magnification (0 = none, 1 = minimal, 2 = less than half of fields fibrotic, 3 = more than half of fields fibrotic, 4 = all fields fibrotic) and the results averaged for each kidney (Fig 4). Four NHP had kidney sections that were not identified as right or left; for those animals one of the kidney scans was chosen at random for each animal. One NHP had a single kidney.

**Fig 5:**
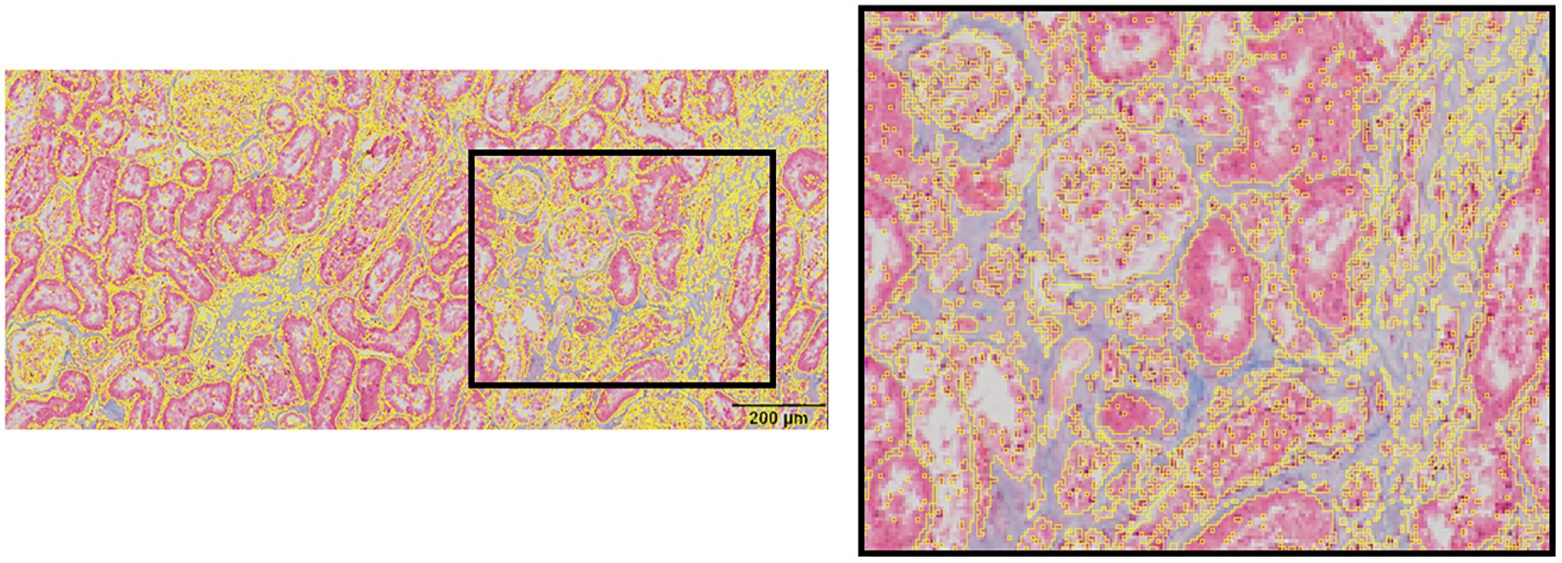
Computer imaging fibrosis scoring. A) Image showing ROIs generated using ImageJ color thresholding to measure the blue area in trichrome stained histology sections. B) Magnified view of the area of the black rectangle in 5A, showing the that these ROI contours include blue areas and exclude pink area and empty space.

Histological fibrosis area was also measured using ImageJ (https://imagej.nih.gov/ij/) color thresholding. Histological sections from the 12 animals that did not receive thorax only irradiation were digitized using Viziopharm (Westminster, CO). Three renal cortex ROIs were measured from each digitized histological section. Large arteries were avoided. Total trichrome stained blue area was measured in Hue/Saturation/Brightness (HSB) colorspace. ROIs were generated by setting the HSB thresholds to include blue pixels and exclude pink pixels and empty space (Fig 5). The total blue area divided by the total area of each ROI was calculated. All ROIs were measured twice by a single masked researcher. The average of the six (three ROIs measured twice) blue area/total area values was calculated for each histological section.

### Statistical analysis

Akaike’s Information Criterion (AIC) was used to compare model fit of cross-sectional area to TSI. Because the CT numbers of the tissue of interest in this study all fell between the values of phantom regions 1 and 2, we examined whether the slopes differed using a Wald test of interaction. The slopes were not significantly different, therefore one equation was fit for phantom ROIs 1 and 2. To normalize each CT number, we shifted the intercept based on the difference between the actual and predicted CT number for the size index and then calculated the corrected CT number using the equation:

$$\text{Corrected}\;\text{CT}\;\text{number}={\text{Measured}\;\text{CT}\;\text{number}}_{\text{i}}+\text{A}\;\text{ln}\left({\text{size}\;\text{index}}_{\text{i}}/\text{mean}\;\text{size}\;\text{index}\right)$$

where Measured CT number_i_ and size index_i_ are the values measured for each animal and mean size index is the mean size index for the study group. The derivation of this equation and the determination of the value of the ln term coefficient A are described in detail in the accompanying supplement.

Descriptive statistics (means, 95% C.I.) were calculated to describe the characteristics of the subjects. Paired t-tests were used to compare fibrosis scores and corrected CT number scores between left and right kidneys in animals with tissue from known sides. Fibrosis was defined as a mean fibrosis score of 1 or greater. Using a generalized estimating equation (GEE) logistic regression model to account for repeated measures (sides) on each animal, fibrosis was regressed on the CT number, and receiver operating characteristic (ROC) analyses were performed to assess the ability of CT to discriminate fibrotic renal cortex from non-fibrotic renal cortex. We chose a GEE model to fit the marginal effect of CT on fibrosis, averaged over the study population [10]. To obtain an estimate of the correlation between methods (clinical scoring of histology, CT, and histological fibrosis area using ImageJ) accounting for the repeated measures by side, a mixed model with random effects was used, treating the method and the side as repeated measures on the animal and including method as a fixed effect to account for the different means by method for all animals, and for animals with fibrosis scores greater than zero.

## Results

All four phantom ROIs exhibit the subject size dependent drop in CT number as subject size increases for both CSA and TSI (Fig 2). TSI explained more variation in CT number than CSA, as evidenced by the lower AIC (AIC = 380.6 for CSA vs 360.5 for TSI), suggesting more of the size dependent CT number variance can be removed by using the TSI than by using CSA as the size index. Therefore, TSI was chosen as the size index for the CT number size correction in this study.

The ten irradiated NHP had undergone 5 to 8 Gy total body irradiation an average of 7.9 (7.0 to 9.0, 95% C.I.) years previously. Their BUN at euthanasia was 24 mg/dl (14 to 40 mg/dl, 95% C.I.). The two age-matched non-irradiated controls had an average BUN of 21 mg/dl at euthanasia and the ten that underwent chest-only irradiation had an average BUN of 16 (13 to 20, 95% C.I.) at euthanasia. These non-irradiated control NHP thus had normal BUN values.

The renal injury had features of chronic radiation nephropathy as have been described in irradiated non-human primates [11,12] with glomerular and tubulo-interstitial scarring that predominated in the cortex. The NHPs that received total body irradiation had mean histological fibrosis scores ranging from 1 to 3.25 and corrected CT numbers ranging from 48.2 to 65.4 HU. The NHPs that received chest-only irradiation had histological kidney fibrosis scores ranging from 0 to 2 and corrected CT numbers ranging from 45.6 to 60.8 HU. The group that received total body irradiation had higher size corrected CT numbers than the group that received chest only irradiation (56.8 HU vs 50.8 HU p<0.01) and had higher fibrosis scores (2.0 vs 0.5, p<0.001). No significant difference was found between left and right kidney histological fibrosis score (mean difference of right minus left = -0.13, p = 0.34) or between left and right corrected kidney CT number (mean difference of right minus left = 0.09 HU, p = 0.90), suggesting that it is reasonable to pool left and right kidneys.

There was a direct correlation of the human observer fibrosis scoring to the adjusted CT scan HUs (r = 0.8, p < 0.0001). There was a direct correlation of the BUN to the fibrosis score and the adjusted CT scan HUs (r = 0.672 p = 0.0001, r = 0.549 p = 0.0004, respectively).

**Fig 6:**
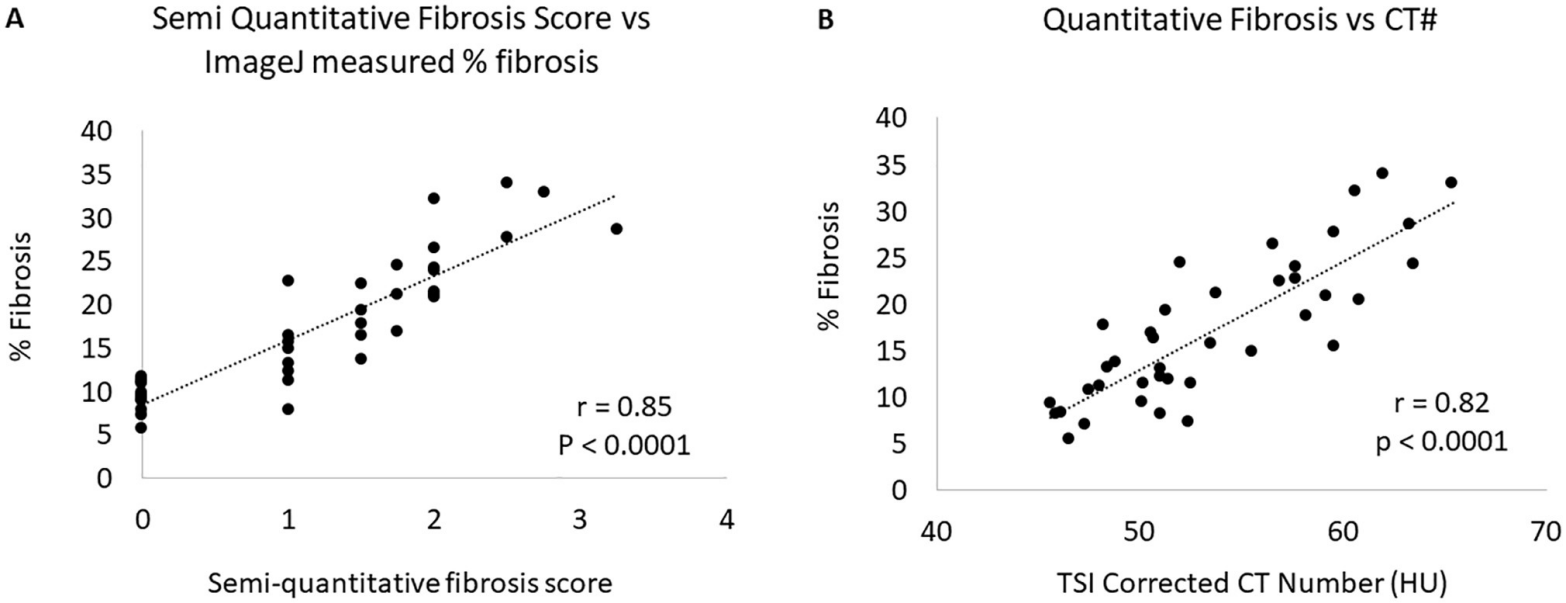
Correlation of tissue fibrosis scoring with size corrected CT HU. A) Quantitative color threshold scoring of blue-stained fibrosis correlates well with semiquantitative human scoring and B) also with size-corrected CT number.

Quantitative ImageJ histological fibrosis scoring correlated very well with human observer histological scoring (r = 0.85, p = 0.0001) and with size corrected CT# (r = 0.82, p<0.0001) (Fig 6).

**Fig 7:**
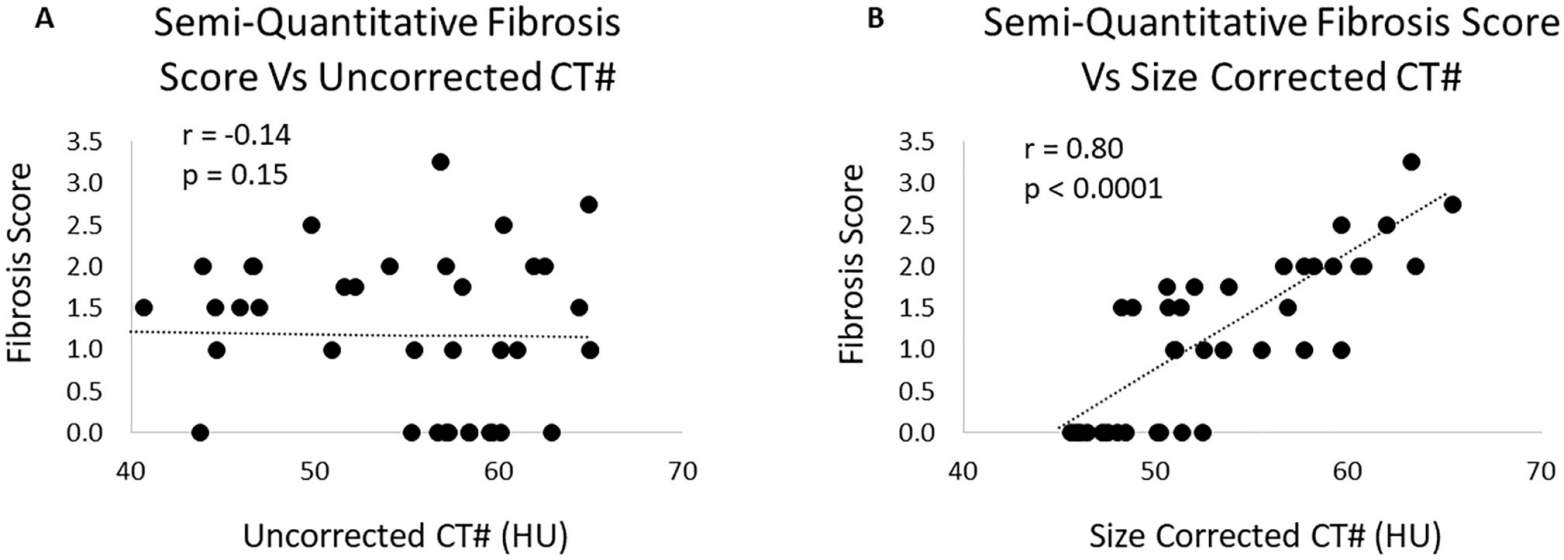
Effect of size adjustment on the correlation of CT HU to tissue fibrosis score. A) The correlation between histology fibrosis scores and uncorrected CT number is obscured by the dependence of CT number on subject size. B) When the CT number is corrected for subject size using the total CT total signal index, there is a strong correlation between histology fibrosis score and CT number.

There was no correlation of the histological fibrosis score to the uncorrected CT HU. But there was an excellent correlation between fibrosis score and corrected CT number at r = 0.80 (Fig 7, p<0.0001). Evaluating only those kidneys with fibrosis > 0, the correlation between fibrosis score and corrected CT number was r = 0.67 (p = 0.001).

**Fig 8:**
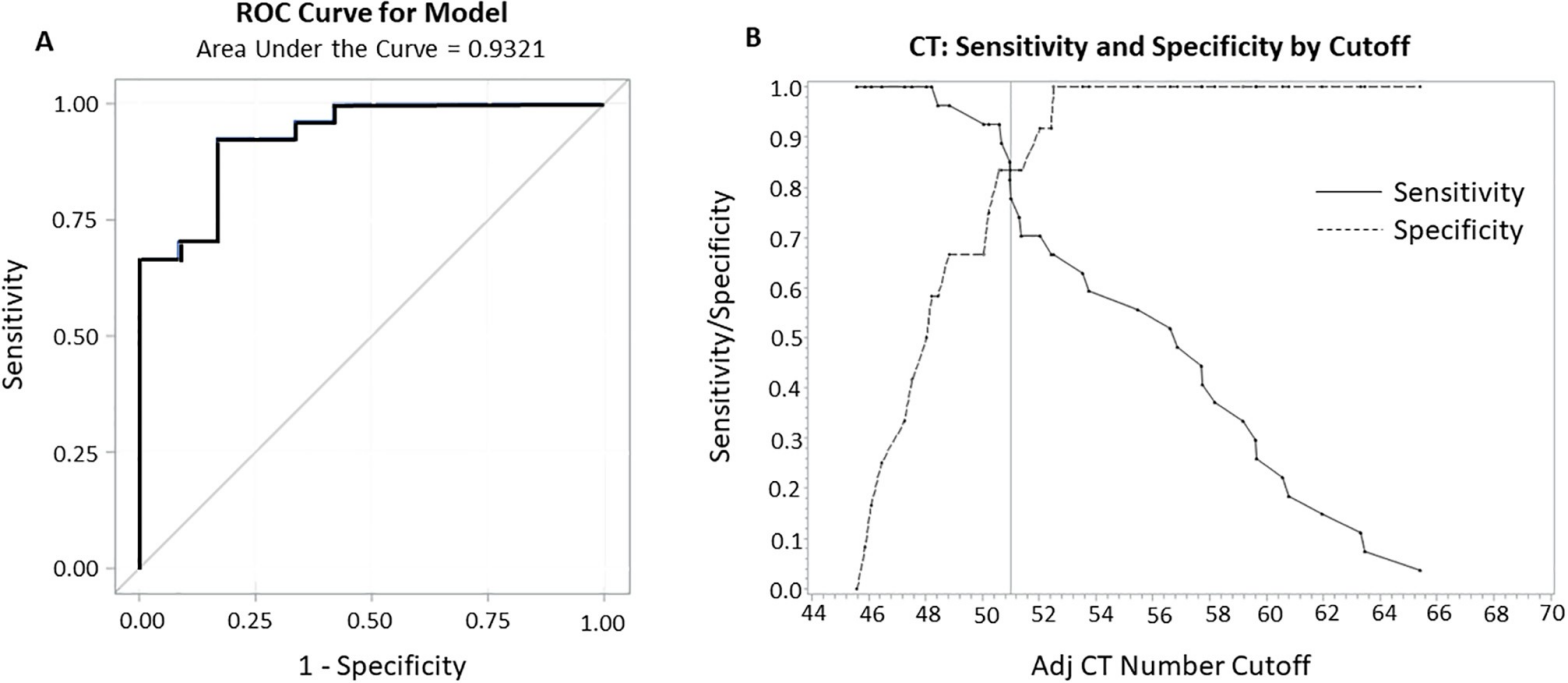
Receiver operating curve, sensitivity, and specificity for CT HU to indicate fibrosis. A) Receiver Operating Characteristic Curve. Showing an AUC of 0.93. B) Graph of Sensitivity and Specificity by cutoff.

The area under ROC curve (AUC) was 0.93, which indicates excellent discrimination of fibrosis from no fibrosis using size corrected CT numbers (Fig 8). With a cutoff CT number value of 51 HU, the specificity was 83% and the sensitivity was 78%. The positive predictive value was calculated at 91%, and the negative predictive value was 63%. When the cutoff was below 48.4, the negative predictive value was 100%; when the cutoff was above 52.5, the positive predictive value was 100%. The overall prevalence of fibrosis was 66%. Results were similar when analyses were repeated with unknown sides excluded.

## Discussion

We show that CT scanning can identify and also quantify renal fibrosis in an NHP model. The area under the curve (AUC) for our CT method is 0.93. This compares well to the AUC for TGF-beta of 0.90, which was identified as the best renal fibrosis biomarker in a recent comprehensive review [13]. Our method yields more than a binary yes/no indication of fibrosis, because the corrected CT number correlates with increasing severity of fibrosis.

Using all data, we can rule out the presence of fibrosis when the corrected CT number was below 48 HU in this high fibrosis prevalence cohort. Having a defined cutoff for size corrected CT number below which there is no fibrosis will enable the timed use of anti-fibrotic agents before fibrosis starts. It will also help to validate blood or urine biomarker candidates that correlate with fibrosis. The very good specificity of the size corrected quantitative CT method is very useful to define a threshold of significant fibrosis. The direct dose response relationship of corrected CT number to increasing degrees of fibrosis may permit detailed study of fibrotic diseases and their progression, not just the yes/no question of fibrosis versus no fibrosis.

It is likely that features of renal parenchymal injury such as cysts or hydronephrosis will affect the CT HU. Cysts were not a frequent feature in the present studies, and were avoided when drawing the ROIs. Hydronephrosis was not seen in these studies.

This unique cohort had two major advantages. First, we had whole kidneys for histological study, which enabled confident grading of whole organ fibrosis. Further, we account for the effect of animal size and associated beam hardening on CT number. This correction is an essential step to enable reliable correlation of fibrosis to the CT number. But we caution against using uncorrected CT number as read by clinical CT in current human practice to grade renal fibrosis. That is because the correlative studies have not been done, and because of the likely effects of patient-size-dependent beam hardening that influence the non-corrected CT number readings.

CT numbers corrected using total CT signal yield an area under the ROC curve of 0.93, which represents an excellent diagnostic tool. We expected total CT signal to be better than CSA as an index of x-rays absorbed, because the total signal is the scanner’s own estimate of the total x-ray energy absorbed. The TSI is a function of not only the area of the material in the slice, but also the mean attenuation coefficient of the material in the slice.

The whole body CT scans used in this study were acquired routinely as a part of a larger radiation study, without knowing what organs would be ultimately studied. Therefore, the QCT phantom was not always present in the slices that included our tissue of interest. The size correction method developed in this study allowed scans that did not have the QCT phantom present in the slice of interest to be used in the quantitative CT study.

The relationship that exists between CT number and subject size can be expected to vary from scanner to scanner. Our exact mathematical models and coefficients cannot be expected to work on a different CT scanner. This study does, however, provide a template of the workflow for a quantitative CT study, using scans from about 10 different sized subjects with a QCT phantom to model a scanner’s residual beam hardening artifact, followed by applying that model to correct CT numbers for subject size using the novel CT subject size index.

We did not look at the dependence of CT number on subject size for scanning protocols with different parameters (e.g. kV, mA, etc.) Future studies can test to see how the models depend on scan parameters and how they vary from machine to machine.

Clinicians familiar with human kidney HU values may be surprised that our corrected CT numbers for renal cortex range from 47 to 64. We normalized our CT numbers to the average size of this cohort. The average subject weight of this cohort was 9.7 kg, which is about 1/7 the weight of a human. It is to be expected that a larger human will have a lower CT number for equally-fibrotic renal tissue with the same absorption coefficient than a smaller NHP due to beam hardening. It is also possible that varying bone mass could distort CT HU values, but our total size index takes this into account.

We used trichrome-stained sections to identify and grade fibrosis. Other methods can be used, including picrosirius red or stains for specific types of collagen. We used two methods to identify and grade fibrosis in the histology, semiquantitative human readings and quantitative color threshold area measurements. A recent review of this topic finds human readings to be the best method for identification of renal fibrosis [14], but there may be lingering concern that a human could be influenced by other visual cues of renal pathology which may affect the evaluation of fibrosis burden. Our second method of quantitative color thresholding cannot be influenced by pathology that does not affect the trichrome color staining. The strong correlation between semiquantitative and quantitative histological scoring affirms the reliability of semiquantitative human scoring of fibrosis in trichrome stained histology sections. The strong correlation between fibrosis burden as determined by quantitative color thresholding and size corrected CT number leads to the conclusion of this study that size corrected CT is correlated with the amount of fibrosis present in the renal cortex.

Renal ultrasound is used to define renal scarring. Increased echogenicity by renal ultrasound correlates with fibrosis but this is reported in a binary, yes/no fashion. In addition, increased echogenicity by ultrasound generally correlates with impaired renal function [15]. The present studies appear to show detection of fibrosis when BUN is only minimally elevated.

Magnetic resonance imaging (MRI) techniques can detect tissue fibrosis indirectly by determination of capillary loss, tissue stiffening by MR elastography, and by detection of changed water movement within the parenchyma [16]. The latter appears to show an inverse and significant dose response relationship between the apparent diffusion coefficient and histological fibrosis. But MRI is a more expensive imaging procedure than CT, and cannot be used in subjects who have metal implants such as pacemakers or artificial joints.

Use of CT to image fibrosis has concerns for increased risk of morbidity, such as causing cancer, in the irradiated subject. The effective absorbed dose values for human CT scans of the abdomen are 5 to 7 mSv (0.5 to 0.7 rem) [17]. This dose is about twice that of the annual average US background dose from natural radiation sources (≈ 3 mSv/yr). But to directly increase mortality from cancer, a single radiation dose must be at least 30 mSv or more [18]. The risk of one or several CT scans is therefore quite low, and possibly even non-existent. In addition, as in the present study, previously-acquired CT scans can be re-purposed for analysis of the relation of fibrosis to subject size corrected CT number.

It is possible that this technique could be adapted to quantify fibrosis in other organs. We chose to focus on renal fibrosis in the present studies. Generally, this technique could be applied to diagnosis or treatment of fibrotic diseases but it requires confirmation before such use.

## Conclusion

Subject size corrected CT number correlates very well with renal parenchymal fibrosis. This imaging biomarker of renal fibrosis will benefit from further study for animal and human use.

## Supporting information

S1 Fig (DOCX)

S1 Data (XLSX)
