# Supplementary material for: Detection and quantification of renal fibrosis by computerized tomography
Source: PLoS One. 2020 Feb 13;15(2):e0228626. doi: 10.1371/journal.pone.0228626 (PMC7018060; doi:10.1371/journal.pone.0228626)
Supplement: S1 Fig — (DOCX) [file pone.0228626.s001.docx]

**Supplement**

Detection of renal fibrosis by computerized tomography

**Quantitative CT Subject Size Correction Using Total CT Signal as Subject Size Index.**

We tested two indexes of subject size: 1) the cross-sectional area (CSA) of tissue and material traversed by x-rays in the slice of interest and 2) the total CT signal index (TSI) in the slice of interest. We expected that a suitable index for animal size to correct for the effects of beam hardening would be an index that correlates with the total x-ray energy absorbed by tissue in the slice of interest. Therefore, CSA should be better than body weight and total body surface area because different parts of the body have different axial cross-sectional areas, and TSI should be better than CSA because it accounts for variable x-ray absorption across all tissue.

**Methods**

Total CT signal was defined as the mean CT number for the entire Field of View (FOV) times the area of the entire FOV of the slice. This product gave a negative number because most of the area of the entire FOV was air, which for our scanner has a CT number of -1024 HU. We subtracted the CT number of air (-1024 HU) from the mean CT number for the entire FOV, before we multiplied by the area of the FOV, so TSI would be a positive number. Our image analysis software (TeraRecon) gives ROI areas in square millimeters, so we divided this product by 10^7^ to give us a total signal index that was between 0 and 10 for our subjects. Our final formula to calculate TSI from the measured FOV area and mean FOV CT signal is:

TSI = (Mean CT#_entire FOV_ – CT#_air_) Area_FOV_ /10^7^  (Equation 1)

We observed a decrease in the CT number of phantom regions of interest (ROIs) as the size of the subject in the scan with the phantom increased. We regressed the CT number on the natural log of CSA for each of the four phantoms, and we also regressed the CT number on the natural log of TSI for each of the four phantoms, using a generalized estimating equation model (GEE) to account for the repeated measures on the animals. For our scanner, the relationship between the predicted CT number as a function of TSI can be expressed as:

Predicted CT number = B – A ln(TSI_i_) (Equation 2)

Where TSI_i_ represents the TSI value for an individual monkey. Akaike’s Information Criterion (AIC) AIC was used to compare model fit of CSA to TSI. Because the CT numbers of interest in this study all fell within the values of phantoms 1 and 2, we examined whether these two phantoms could be combined by testing whether the slopes differed using a Wald test of interaction. The slopes were not significantly different, and therefore one equation was fit for phantoms 1 and 2. To normalize each CT number, we shifted the intercept based on the difference between the actual and predicted CT number for the TSI value, and then calculated the corrected CT number using the equation:

Corrected CT number = {B + (Measured CT number - Predicted CT number)} – A ln(mean TSI) (Equation 3)

where the average TSI value for the group is used for mean TSI. Substituting Equation 2 into Equation 3, it can be seen that

Corrected CT number = Measured CT number + A ln (TSI_i_/mean TSI) (Equation 4).

**Results**

All four densities in the phantom exhibit the subject size dependent drop in CT number as subject size increases for both CSA and TSI (Figure 2 in the paper). TSI explained more size dependent variation in CT number than area, as evidenced by the lower AIC (AIC=380.6 for CSA vs 360.5 for TSI), suggesting more of the size dependent CT number variance can be removed by using the TSI than by using CSA as the size index.

The constant coefficient B in equation 2 represents a normalized CT number, the value of the CT number when TSI is equal to 1. Combining phantoms 1 and 2 resulted in the prediction equation:

Predicted CT number = 57.97 + -19.86*ln (TSI) (Equation 5)

We then used this model to normalize each renal cortex measurement to the average TSI for the entire cohort. Below is a sample size correction calculation for a subject that had a TSI of 2.2255 and a mean uncorrected renal cortex CT number of 50.9. Equation 3 can be used to calculate the expected CT# of a renal cortex in an animal with this size:

Predicted CT number = 57.97 + -19.86 * {ln(2.2255)} = 42.08 (Equation 6)

That means this particular kidney is 50.9 – 42.08 = 8.82 HU more intense than predicted. The linearized equation that describes this kidney’s CT# vs {ln(TSI)} is:

Corrected CT number = (57.97 + 8.82) -19.86*{ln(mean TSI)} (Equation 7)

The average TSI of the group is 1.7667, leading to a corrected CT number of 55.49 for the subject (Figure S1).

(Alternatively the equation Corrected CT number = 50.9 + 19.86*{ln(2.2255/1.7667)}=55.49 could be used.)

**Discussion**

Our final corrected CT numbers are higher than might be expected for human kidney tissue. The subjects are non-human primates with an average body weight of 9.7 kg, which is about 1/7^th^ the body weight of a human. The effect of beam hardening is responsible for making the CT numbers of tissue of smaller animals higher than the same tissue in humans. Clinical scanners employ a variety of proprietary methods to reduce beam hardening artifacts. The mathematical relationships that we observed and the coefficient values that work for our scanner and scanning protocol cannot be expected to work exactly the same for all CT scanners. However, our approach can be performed for different scanners.

Figure S1


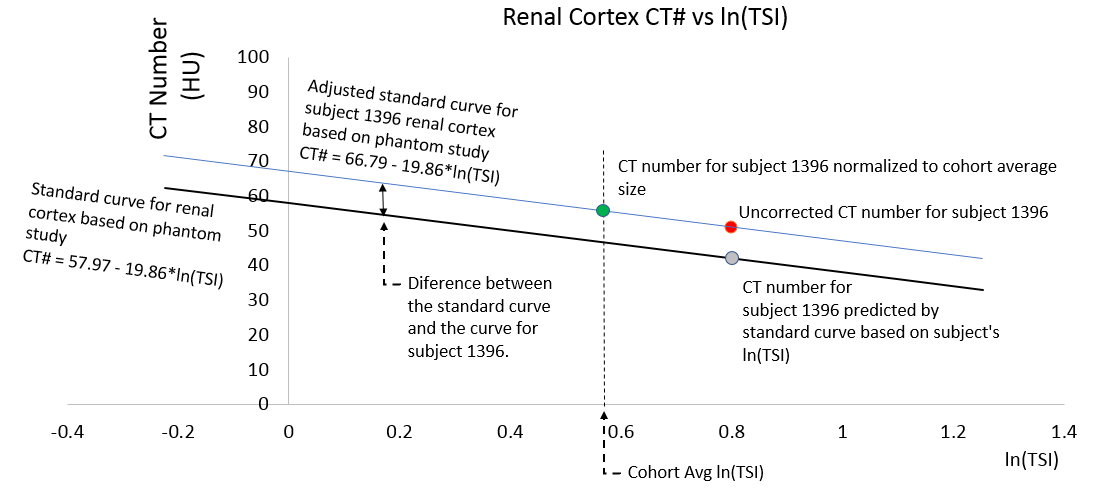


Figure S1 Graphical representation of the quantities involved in the size correction. The red dot represents the uncorrected CT number and the ln(TSI) of subject 1396. The gray dot is the CT number predicted by the model for renal cortex tissue. The standard curve is shifted up or down to pass through the uncorrected CT number of the subject. Then the shifted standard curve is used to calculate the corrected CT number of the subject at the cohort average ln(TSI).
